# A Ku-Band Compact Offset Cylindrical Reflector Antenna with High Gain for Low-Earth Orbit Sensing Applications

**DOI:** 10.3390/s24237535

**Published:** 2024-11-26

**Authors:** Bashar A. F. Esmail, Dustin Isleifson, Lotfollah Shafai

**Affiliations:** 1 Department of Electrical and Computer Engineering, University of Manitoba, Winnipeg, MB R3T 5V6, Canada; bashar.esmail@umanitoba.ca (B.A.F.E.); lot.shafai@umanitoba.ca (L.S.); 2 Centre for Earth Observation Science (CEOS), University of Manitoba, Winnipeg, MB R3T 5V6, Canada

**Keywords:** CubeSats, cylindrical reflector, high-gain antenna, low-Earth orbit, microstrip patch antenna

## Abstract

The rise of CubeSats has unlocked opportunities for cutting-edge space missions with reduced costs and accelerated development timelines. CubeSats necessitate a high-gain antenna that can fit within a tightly confined space. This paper is primarily concerned with designing a compact Ku-band offset cylindrical reflector antenna for a CubeSat-based Earth Observation mission, with the goal of monitoring Arctic snow and sea ice. The development of a Ku-band offset cylindrical reflector, with a compact aperture of 110 × 149 mm^2^ (6.3*λ* × 8.5*λ*), is described alongside a patch array feed consisting of 2 × 8 elements. The patch array feed is designed using a lightweight Rogers substrate and is utilized to test the reflector. Adopting an offset configuration helped prevent gain loss due to feed blockage. Analyzing the reflector antenna, including the feed, thorough simulations and measurements indicates that achieving a gain of 25 dBi and an aperture efficiency of 52% at 17.2 GHz is attainable. The reflector’s cylindrical shape and compact size facilitate the design of a simple mechanism for reflector deployment, enabling the antenna to be stored within 1U. The array feed and reflector antenna have been fabricated and tested, demonstrating good consistency between the simulation and measurement outcomes.

## 1. Introduction

Synthetic aperture radar (SAR) interferometry and other satellite remote sensing instruments are pivotal for studying changes in Earth’s surface topography or deformation. These tools are highly effective for mapping the topography of Earth’s land, ice, and even sea surfaces. Leveraging deformation measurements is essential for the management of global natural resources and mitigation of hazards, providing crucial information for scientists to understand the impacts and rate of climate change [1]. The Arctic is rapidly exhibiting signs of global climate change [2]. The transition from thick perennial sea ice to a seasonally ice-free Arctic Ocean has major consequences for sustainable development, transportation, and communities in Arctic regions [3,4]. Designing a small satellite for such a mission is essential for studying changes in sea ice extent, type, and coverage in the Arctic. Currently, there is a growing trend in utilizing small spacecraft and their constellations to address pressing issues concerning Earth observation from space [5]. A CubeSat is a standardized small spacecraft concept introduced at the end of the previous century [6]. Within CubeSats, the antenna plays a pivotal role as part of the radio frequency (RF) front end, facilitating the transmission of RF waves between space and Earth. Satellite antennas are required to facilitate communication across various frequency bands, including high-speed links like S-band, X-band, and Ku-band for data downlink, as well as low data rate links such as Very High Frequency (VHF) and Ultra High Frequency (UHF) for telemetry and control data [7,8]. Compact antennas must align with spacecraft specs and meet mission requirements. Proposed space mission antennas include the monopole, dipole, slot, patch, helical, reflectarray, and reflector types [9,10,11,12]. The first four are often chosen due to their light weight, compact size, and ease of fabrication. Nevertheless, their performance in terms of gain tends to be moderate, a crucial aspect in the antenna design process for space missions. Helix antennas offer moderate gain along with inherent circular polarization and broad bandwidth. Nevertheless, their deployment mechanism presents significant design hurdles, particularly when the antenna length surpasses the satellite’s dimensions. Reflectarray antennas offer the advantages of being lightweight, relatively inexpensive, and foldable into panels for efficient stowage. However, they are limited by narrow bandwidths (<10%), and the maximum gain is constrained by the number of panels feasible for folding into a CubeSat [13,14]. Reflector antennas, like the parabolic and cylindrical types, offer high gain, but their large structures pose a challenge for fitting them into SmallSats. Deployable systems offer a solution to this dilemma by allowing the large antenna size to be stowed in a compact space. These systems allow antennas to be stowed inside the CubeSats during launch and then deployed once they are in orbit. Antenna systems, as in [15,16], based on reflectors for high-frequency bands, were achieved with meshed or inflatable antennas. Although these deployable systems can make optimum use of the footprint in CubeSats, the complexity of deployable mechanisms may limit their commercial viability.

Cylindrical parabolic reflectors are widely employed in various applications, especially in satellite communication systems, where high-gain antennas are essential. They necessitate focus from a line source rather than a single point source. Various feed lines have been suggested to illuminate the cylindrical reflector, such as arrays of horns and patch antenna arrays [17,18]. The former is larger in mass and profile compared to the planar array structure, which provides a lightweight and low-profile design, essential factors in CubeSats design. The use of patch antenna arrays to feed cylindrical reflectors is advantageous in radar applications [19]. Few studies in the literature have focused on the design of cylindrical reflector antennas for space applications [18,20,21]. The offset parabolic-cylindrical reflector, detailed in [18], operates at a Ku-band of 13 GHz. Its feed source is an 8 × 4 patch array antenna, and the aperture dimensions are 50 cm × 50 cm (21.7*λ* × 21.7*λ*). The resulting gain is recorded as 26.85 dBi. The authors of [20] introduced a deployable cylindrical reflector designed for low-Earth orbit satellites. Operating at 5.357 GHz, the antenna achieves a high gain of 42 dBi. It employs a 1 × 32 element patch array feed to illuminate the large cylindrical aperture measuring 7 m × 1.5 m (125*λ* × 26.8*λ*). Despite its high gain, the structure is large, posing challenges in stowage to accommodate it within SmallSats. In [21], a linear feed array comprising 12 elements is employed to feed the cylindrical reflector with aperture dimensions of 140 cm × 50 cm (10.2*λ* × 3.9*λ*). The structure yields a medium gain of 11.2 dBi at 2.17 GHz. However, the gain and size of the reflector may not be appealing for CubeSats.

**Figure 1:**
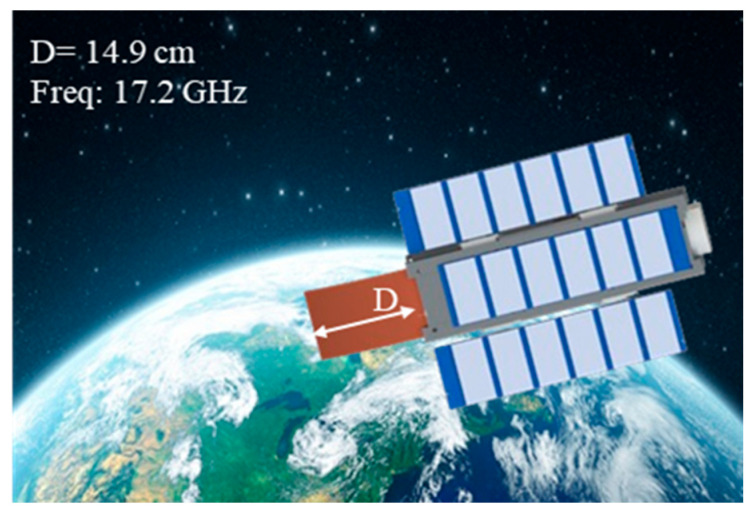
6U CubeSat employing the proposed deployable cylindrical reflector antenna. The antenna is designed to fit within 1U (10 × 10 × 10 cm^3^) [24].

Although C-band radar measurements have traditionally been utilized in sea ice remote sensing, research indicates that other frequency bands, notably the Ku-band, are effective in discerning snow and ice thickness [22,23]. An effective Ku-band antenna must have high gain, low cross-polarization, a narrow beamwidth, and sufficient bandwidth to give usable on-ground image resolution. Few studies have addressed the use of cylindrical reflectors for space applications, and none have specifically proposed them for CubeSats. This paper outlines the design, construction, and testing of an offset cylindrical reflector antenna intended for low-Earth orbit observation, primarily focusing on monitoring Arctic snow and sea ice (shown in Figure 1) [24]. The microwave emissions from the Earth are collected by the antenna and is fed into the front-end of the radiometer. The brightness temperature measured by the radiometer is used to distinguish between sea ice, open water, and other features. The feed comprises an antenna array operating at a frequency of 17.2 GHz. The ratio of focal length to diameter of cylindrical reflector (*F*/*D*) is 0.65, chosen to achieve the desired reflector response while also maintaining the structural stiffness of the antenna. The feed is located along the axis of the cylindrical reflector, positioned at a distance *F* = 98 mm, and angled by 45° to illuminate the reflector. The developed reflector achieves a high gain of 25 dBi. Both the reflector and feed have been fabricated and measured, confirming a high degree of agreement between simulation and measured outcomes. The remainder of this paper is structured as follows: Section 2 offers comprehensive details on the design of the cylindrical reflector antenna, and the array feed design and its experimental validation. Section 3 presents the experimental results. Section 4 outlines the proposed deployable mechanism, while Section 5 concludes the paper.

## 2. Antenna Design

### 2.1. Antenna Specifications

The antenna specifications are determined by operational requirements for a constellation of SmallSats tasked with monitoring Arctic snow and sea ice. The antenna specifications are presented in Table 1, which are based on a 550 km orbit. The proposed antenna is optimized at 17.2 GHz over a bandwidth of 200 MHz. To minimize the complexity of the deployment mechanism, an offset cylindrical reflector was selected. The feed offers linear polarization, providing the desired bandwidth with over 20 dB return loss at 17.2 GHz. With a simple deployable mechanism, it is possible to stow the reflector within 1U (10 × 10 × 10 cm^3^). Antenna production expenses need to account for only a small fraction of the total mission budget, similar to the scenario with composite antennas frequently employed in satellites and communication systems.

### 2.2. Patch Array Feed

**Figure 2:**
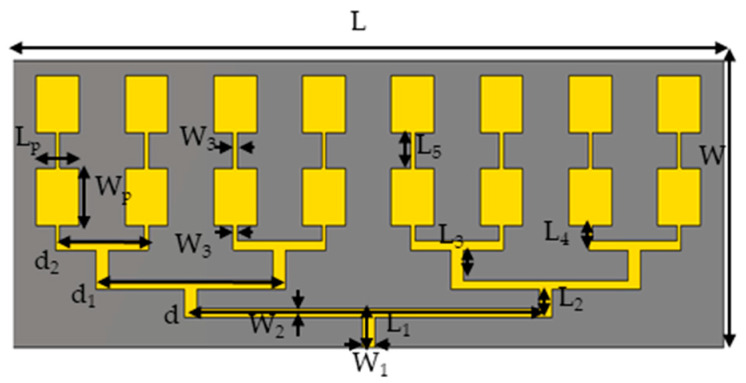
The configuration of the 2 × 8 array feed.

An array of microstrip patches is used to illuminate the offset reflector. The selected setup consists of a 2 × 8 patch antenna array, illustrated in Figure 2. To prevent a grating lobe in the scanning plane of the reflector, an array spacing of d = 0.4*λ* (*λ* is the free space wavelength) is adopted. The substrate used is Rogers RT5880, featuring a dielectric constant of 2.2, a loss tangent of 0.0009, and a thickness of 0.508 mm. This substrate was selected for its low dielectric constant and minimal dielectric loss, offering an ideal balance between high performance (in terms of bandwidth and radiation characteristics) and the mission’s size and mass constraints. Its low contribution to overall losses is primarily due to the minimal dielectric loss. The geometrical parameters of the antenna array are presented in Table 2. Rectangular patches receive power from microstrip power-dividing network on the same layer. The main line consists of a microstrip line width of W_1_, with a characteristic impedance of 50 Ω. A 50 ohm coaxial connector mounted on the edge is utilized to excite the array feed. Designed with impedances of 70.7 Ω and 100 Ω, the widths W_2_ and W_3_ support the necessary impedance transformation across the feed network. The lengths L_1_ through L_5_ along with other parameters, were optimized to achieve a reflection coefficient, S_11_, of less than −20 dB at 17.2 GHz, ensuring the desired radiation characteristics for the array patch.

It is important to mention that while the array could be further optimized, the primary aim here was to design a compact and lightweight array for testing the cylindrical reflector performance, as elaborated upon in Section 2.3. The simulation of the feed array was carried out using Computer Simulation Technology Microwave Studio (CST MWS).

**Figure 3:**
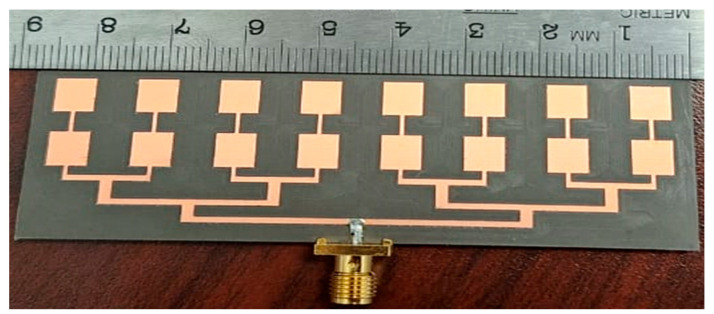
The fabricated array feed.

**Figure 4:**
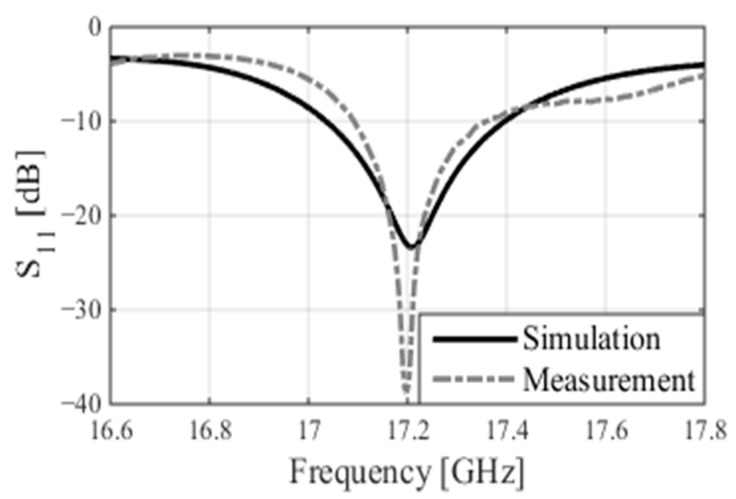
The simulated and measured reflection coefficients of the feed.

**Figure 5:**
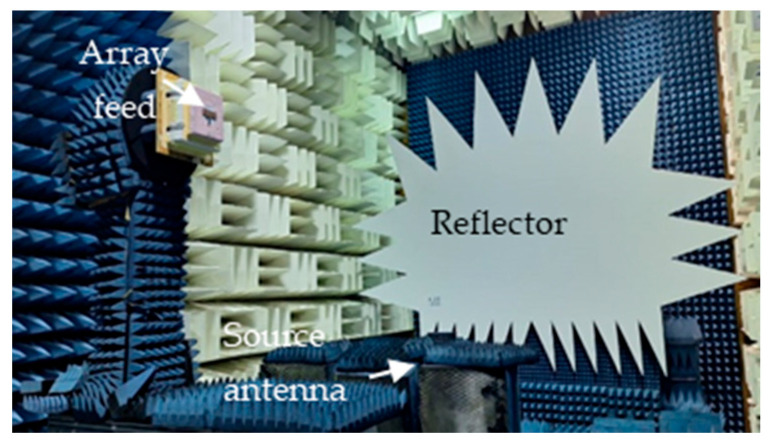
Radiation pattern measurement setup.

**Figure 6:**
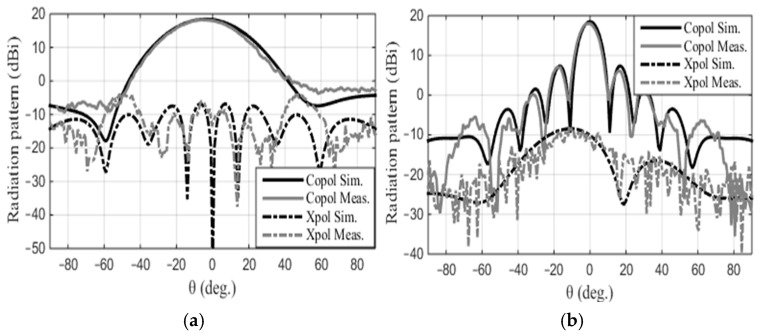
The simulated and measured radiation patterns of the feed at 17.2 GHz: (**a**) E-plane and (**b**) H-plane.

**Figure 7:**
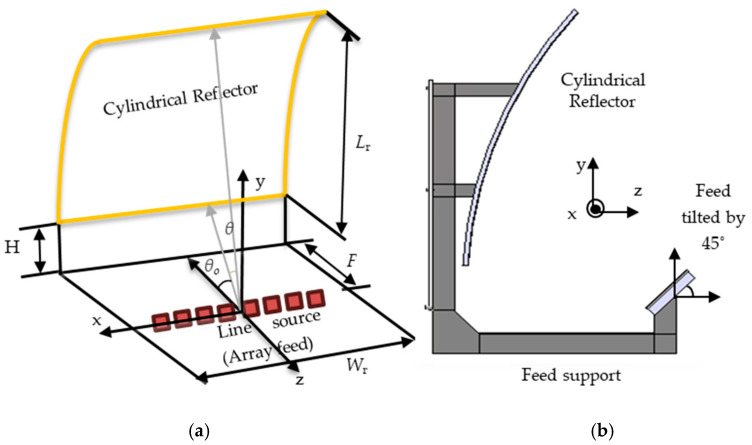
The offset cylindrical reflector system: (**a**) optical specifications for the offset-fed cylindrical reflector with the key parameters (the dimensions are *W*_r_ = 110 mm, *L*_r_ = 149 mm, *F*= 98 mm, and *H* = 13 mm) and (**b**) the cross-section of the reflector system.

The patch array was manufactured using the MiniMill machine at the Machine Shop, Electrical Department, University of Manitoba, as depicted in Figure 3. The feed measurement was conducted at the antenna testing facilities at the University of Manitoba. The measured reflection coefficient, S_11_, is shown in Figure 4 and closely aligns with the simulated result. The feed operates at 17.2 GHz with a bandwidth ranging from 17.02 GHz to 17.4 GHz. The measured bandwidth falls within the range of 17.08 GHz to 17.38 GHz. The slight discrepancy in bandwidth between the simulation and measurement could be attributed to fabrication tolerances and cable losses. The reflection coefficient is below −20 dB for both simulation and measurement data. Radiation pattern measurements were conducted in the anechoic chamber, as illustrated in Figure 5. The co- and cross-polarization radiation patterns of the feed, in the E- and H-planes, at 17.2 GHz are depicted in Figure 6a,b. There is excellent agreement between the simulation and measurement results for both polarizations. The addition of the second row of the array serves to narrow the beamwidth in the plane of the parabola and subsequently provides proper illumination of the reflector cut (along the *y*-axis, as shown in Figure 7). After a detailed analysis, a spacing of L_5_ = 3.62 mm was found to be the optimal distance between the two patch rows, maintaining an operating frequency of 17.2 GHz and delivering the desired radiation characteristics. The cross-polarization level remains under 25.5 dB in both the E-plane and H-plane. While the co-polarization outcomes closely align with the simulation results in the E-plane and H-plane, there exists a minor deviation in cross-polarization. This incongruity may stem from factors like manufacturing tolerances, cable losses, and feed misalignment within the anechoic chamber. The measured gain of the feed at 17.2 GHz is 18.16 dBi, indicating a gain loss of −0.24 dB compared to the simulated gain of 18.4 dBi.

### 2.3. Cylindrical Reflector Antenna

**Figure 8:**
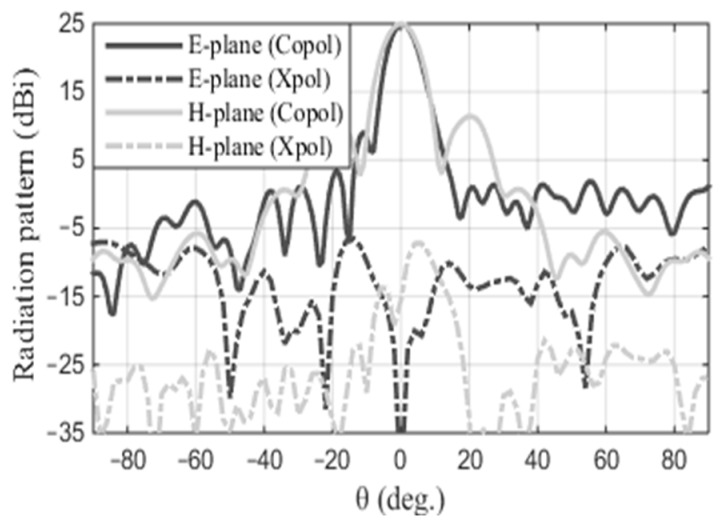
The normalized E- and H-plane radiation patterns at 17.2 GHz.

The reflector is a singly curved offset parabolic cylinder, measuring 110 mm in width and 149 mm in length (equivalent to 6.3*λ* × 8.5*λ*, where *λ* represents the free space wavelength at the center frequency of 17.2 GHz). The arrangement of the offset cylindrical reflector system, along with the feed patch array, is illustrated in Figure 7. The illustration includes details of the geometric parameters and coordinate system. Due to its cylindrical shape, the antenna has a focal line that necessitates a line source rather than a point source to provide proper illumination. The 2 × 8 patch antenna array serves as the feed for the reflector antenna and is positioned along the focal line of the reflector. The antenna aperture is rectangular-shaped, with dimensions of 6.3*λ* × 8.5*λ* and a focal length of *F* = 98 mm (*F*/*D* = 0.65). The clearance height is *H* = 13 mm (cf. Figure 7a). The feed should exhibit a proper pattern in the parabolic cut to provide illumination along the cylindrical axis (*y*-axis). Additionally, ensuring that the feed source is lightweight is crucial to meet potential launching constraints. This can be achieved by designing the array feed on lightweight substrates such as Rogers, as discussed in Section 2.2. The feed is offset and tilted by 45°, as illustrated in Figure 7b, to illuminate the reflector. An offset configuration was employed to prevent gain loss resulting from feed blockage. In this setup, the feed remains along the axis of the reflector, but the reflector itself is cut halfway as shown in Figure 7a. The feed with two rows ensures proper illumination of the reflector cut along the *y*-axis. Minimizing taper and spillover losses required optimizing the feed to achieve a minimum feed taper of −10 dB at 33° (cf. Figure 6a). Proper adjustment of the feed position and its tilting angle enables the achievement of maximum directivity. The maximum directivity can be calculated by

(1) $$D_{max}=10\;\log_{10}\;(\frac{4\pi }{\lambda^{2}})A_{P}$$

where *A_p_* = *L_r_* × *W_r_* is the physical area (the aperture dimensions). The maximum calculated directivity, as per (1), is 28.3 dBi. However, the simulated directivity is 25.4 dBi, resulting in an efficiency of 52%. The discrepancy of 2.9 dB is attributed to taper and spillover losses, along with feed and mismatch losses. When considering the ideal reflector, the overall efficiency *ϵ*= *ϵ_T_ · ϵ_S_* can theoretically reach up to 81% (i.e., −1 dB) [25]. This efficiency is derived from the product of taper efficiency (*ϵ_T_*) and spillover efficiency (*ϵ_S_*), the two primary contributors to aperture efficiency. The taper and spillover losses exhibit a slight increase compared to the ideal scenario. The simulated radiation patterns in both the E- and H-planes at 17.2 GHz are depicted in Figure 8. The simulated half-power beamwidth (HPBW) in the E- and H-planes are 8° and 9.6°, respectively. In the E-plane, the sidelobe level (SLL) is −16 dB, whereas in the H-plane, it is −14 dB. Additionally, the cross-polarization level is below −30 dB in both cuts. It is worth mentioning that the simulated radiation patterns exclude the blockage effects caused by the feed support. Additionally, the cross-polarization shows as null at the boresight, demonstrating the precise alignment of the feed on the reflector.

## 3. Reflector Experimental Validation

**Figure 9:**
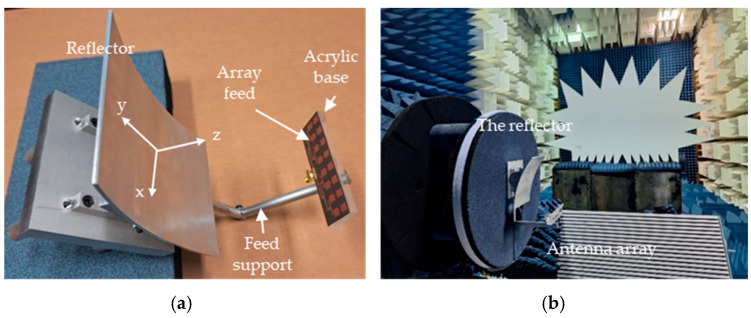
(**a**) The antenna system prototype includes the cylindrical reflector and the array feed and (**b**) the reflector system in the antenna test facility.

**Figure 10:**
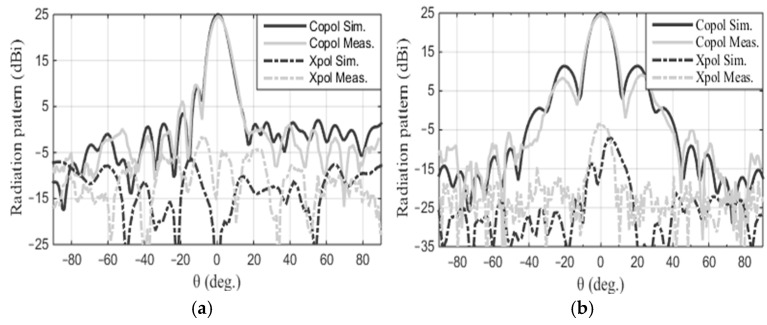
Measured and calculated radiation patterns of the cylindrical reflector at 17.2 GHz: (**a**) E-plane and (**b**) H-plane.

The selected CubeSat antenna is designated for utilization in dual polarizations for remote sensing applications. Its satisfactory performance, especially in cross-polarization, is essential in both polarizations. Since the selected feed support is along the *z*-axis of the reflector, the vertical polarization of the feed excites strong currents on its surface. These currents radiate a linearly polarized wave with vertical polarization, which manifests as a strong cross-polarization on the reflector axis. For horizontal polarization of the feed, the currents on the feed support will be much lower, and the adverse effects on the cross-polarization would not exist. For this reason, the current study was conducted for vertical polarization of the feed to determine its worst case performance. The test antenna consists of the prototype reflector, as described in Figure 9a, along with the feed array discussed in Section 2.2. The reflector is made of aluminum with a thickness of 2 mm, chosen to meet the rigorous demands of space missions. The reflector main beam direction aligns along the *z*-axis. An aluminum metal rod provides support for the feed, which is horizontal in Figure 9a and terminates with an acrylic base. The objective of these measurements is to confirm that the radiation performance of the reflector matches the simulations. It is essential to highlight that the experimental model incorporates the feed support, which was not accounted for in the simulation. This support scatters the feed radiation, which becomes potent as a result of the strong current generated on the supporting rod by the feed polarization. This, in turn, leads to pronounced cross-polarization in the H-plane. Both the reflector and feed were set up in the measurement facility, as depicted in Figure 9b, where the feed polarization is vertical. The antenna underwent testing at the antenna and microwave lab, University of Manitoba, which is equipped for radiation measurements up to 40 GHz. The radiation pattern was assessed in both the elevation and azimuth planes at 17.2 GHz. The measured co- and cross-polarization radiation patterns in both the E- and H-planes at 17.2 GHz are depicted in Figure 10, showing a good match with the simulated patterns, specifically for the co-polarization. There is an evident discrepancy in the cross-polarization data, particularly in the E-plane, which can be attributed to the same factors mentioned in the feed radiation pattern measurement, along with inaccuracies in the assembly of the reflector. The cross-polarization in the E-plane shows as null on the boresigh, a good indication of the symmetric feed alignment. It however has a peak in the H-plane caused primarily by the scattered field of the feed support, and cross-polarization of the feed itself, as shown in Figure 6b. The measured HPBW in the E- and H-planes are 8.2° and 9.7°, respectively. In the E-plane, the measured SLL is −15.2 dB, while in the H-plane, it is −16 dB. The measured cross-polarization level remains below −26 dB for both measured and calculated data. The antenna’s gain offers a comprehensive evaluation of its performance, encompassing all sources of losses within the system. The measured gain of the reflector is 24.5 dBi, closely matching the simulated value of 25 dBi. The measurement discrepancy of −0.5 dB is attributed to various factors, such as assembling inaccuracies, potential alignment inaccuracies, calibrating errors, and uncertainties in measurements. Table 3 presents the measured gain, cross-polarization, and SLL for both cuts.

## 4. Proposed Deployment of the Reflector

**Figure 11:**
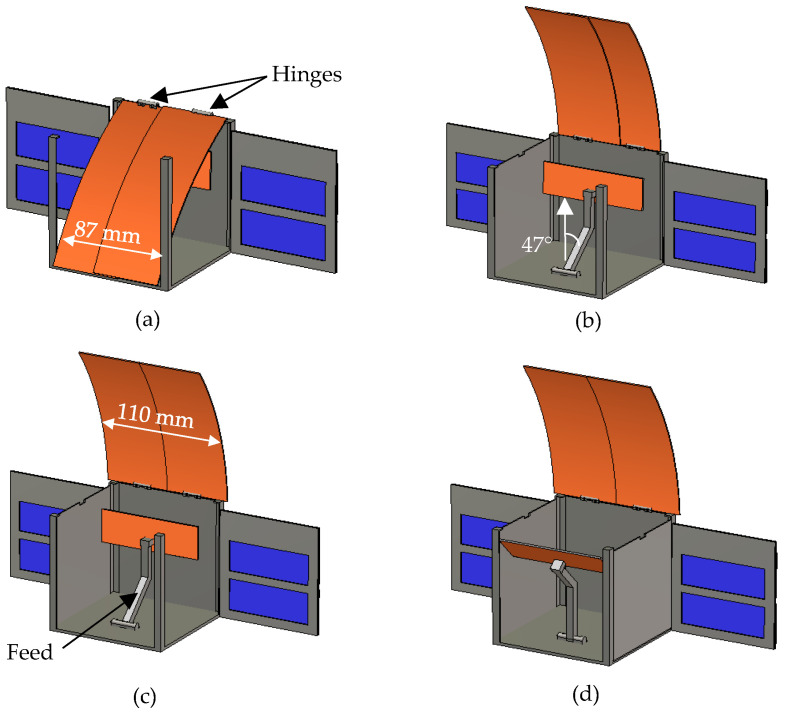
The deployment transitions through the stages of (**a**) fully stowed, (**b**) and (**c**) mid-deployment, and (**d**) fully deployed.

Accommodating a large-scale reflector antenna within 1U is challenging and involves intricate coordination between RF and mechanical design. Parabolic reflector deployment, as in [26], necessitates a significant number of ribs to uphold a reasonable deviation from an ideal surface. The gain loss of such reflectors is predominantly influenced by the number of ribs. Additionally, positioning such a large number of ribs near the reflector can present a complex task and increases the risk of rib jamming during deployment. Thanks to the cylindrical shape and the compact dimensions of the proposed design, achieving the deployable mechanism entails less complexity in contrast to other intricate approaches [26,27]. The proposed deployable mechanism is depicted in Figure 11. This mechanism allows the antenna to be stowed within 1U. To accomplish deployment, the top wall and one of the side walls need to be removed. The sequence from full stowage to full deployment is depicted in Figure 11a through Figure 11d. The fully stowed antenna is illustrated in Figure 11a, with both the feed and reflector contained within a 1U space. The reflector is segmented into two equal parts linked to the CubeSat wall via hinges. To allow the sections to slide over each other, the left part of the reflector is nudged forward, reducing the reflector width to 87 mm to prevent any contact with the 1U walls and rails (standard CubeSat rails measure 6 × 6 mm^2^). The beginning of the full deployment process involves rotating the reflector upward out of the 1U at a 112° angle, as shown in Figure 11b. The correct positioning of the feed to illuminate the reflector is ensured by a support connected to the 1U base. When stowed, it rotates 47° towards the reflector, allowing ample space for the reflector to be stored within the 1U, as demonstrated in Figure 11b. In Figure 11c, the two parts of the reflector slide over each other, and then, the left part is pushed back to its original position, reestablishing the reflector’s full width of 110 mm. The final step involves rotating the feed by 47° away from the reflector to facilitate full deployment, as depicted in Figure 11d. There is available space at the bottom, near each side of the 1U unit, which can be used to house the power source without interfering with the deployment mechanism or compromising the reflector’s performance.

### Future Work

The deployable mechanism represents a foundational concept within this work, serving as a fundamental idea that requires implementation and subsequent testing to validate its functionality and effectiveness. Continual work is dedicated to translating this concept into reality and conducting testing to evaluate its performance. Additionally, the current endeavor will explore a multi-phase center offset reflector antenna [28], employing a unified hardware system to electronically adjust the antenna’s phase center location.

## 5. Conclusions

This paper presents the outcomes of a recent investigation focusing on the development of an offset cylinder reflector antenna, specifically designed to fulfill the needs of low-Earth orbit science missions, with a primary focus on monitoring Arctic snow and sea ice. The primary aim of this work is to devise, construct, and evaluate an offset cylindrical reflector antenna designed to operate at a Ku-band frequency of 17.2 GHz. The reflector features a compact aperture size of 6.3*λ* × 8.5*λ* and a focal length of *F* = 98 mm (*F*/*D* = 0.65). Employing a lightweight 2 × 8 element patch array feed to illuminate the reflector, the antenna achieves a gain of 25 dBi and an aperture efficiency of 52% at 17.2 GHz. The mechanism for reflector deployment was proposed, allowing the antenna to be stored within a 1U space. The array feed has been fabricated and tested, demonstrating favorable agreement across all performance metrics. Furthermore, the reflector system has been manufactured and subjected to testing, yielding commendable gain measurements and radiation pattern characteristics.

## Figures and Tables

**Table 1:** Reflector antenna specifications.

Parameter	Value	Parameter	Value
Frequency	17.2 GHz	Return loss	20 dB
Polarization	Linear	Antenna gain	25 dBi
Bandwidth	200 MHz	Stowed	10 × 10 × 10 cm^3^

**Table 2:** Array antenna fed key parameters.

*L*	*W*	*L*_P_	*W*_P_	*W*_1_	*W*_2_	*W*_3_	*d*	*d*_1_	*d*_2_	*L*_1_	*L*_2_	*L*_3_	*L*_4_	*L*_5_
88	29	5.3	5.1	1.5	0.95	0.47	45	23.6	11.5	3.1	2.9	3.1	2.5	3.62

**Table 3:** Measurement data.

Plane	Gain (dBi)		Xpol (dB)		SLL (dB)		HPBW	
	Sim.	Meas.	Sim.	Meas.	Sim.	Meas.	Sim.	Meas.
E	25	24.5	−31.5	−26.6	−16	−15.4	8°	8.2°
H	25	24.47	−32.2	−28.1	−14	−16	9.6°	9.7°

## Data Availability

All data are available in the manuscript.
